# Structural and catalytic analysis of two diverse uridine phosphorylases in *Phytophthora capsici*

**DOI:** 10.1038/s41598-020-65935-9

**Published:** 2020-06-03

**Authors:** Cancan Yang, Jing Li, Zhenling Huang, Xuefa Zhang, Xiaolei Gao, Chunyuang Zhu, Paul F. Morris, XiuGuo Zhang

**Affiliations:** 1 Shandong Provincial Key Laboratory for Biology of Vegetable Diseases and Insect Pests, College of Plant Protection, Shandong Agricultural University, Tai’an, 271000 China; 2 Department of Biological Sciences, Bowling Green State University, Bowling Green, OH 43403 USA

**Keywords:** Enzyme mechanisms, Enzymes, Proteins

## Abstract

Uridine phosphorylase (UP) is a key enzyme of pyrimidine salvage pathways that enables the recycling of endogenous or exogenous-supplied pyrimidines and plays an important intracellular metabolic role. Here, we biochemically and structurally characterized two evolutionarily divergent uridine phosphorylases, PcUP1 and PcUP2 from the oomycete pathogen *Phytophthora capsici*. Our analysis of other oomycete genomes revealed that both uridine phosphorylases are present in *Phytophthora* and *Pythium* genomes, but only *UP2* is seen in *Saprolegnia spp*. which are basal members of the oomycetes. Moreover, uridine phosphorylases are not found in obligate oomycete pathogens such as *Hyaloperonospora arabidopsidis* and *Albugo spp. PcUP*1 and *PcUP2* are upregulated 300 and 500 fold respectively, within 90 min after infection of pepper leaves. The crystal structures of PcUP1 in ligand-free and in complex with uracil/ribose-1-phosphate, 2′-deoxyuridine/phosphate and thymidine/phosphate were analyzed. Crystal structure of this uridine phosphorylase showed strict conservation of key residues in the binding pocket. Structure analysis of PcUP1 with bound ligands, and site-directed mutagenesis of key residues provide additional support for the “push-pull” model of catalysis. Our study highlights the importance of pyrimidine salvage during the earliest stages of infection.

## Introduction

*Phytophthora capsici*, one of the most destructive plant pathogens of *Phytophthora spp*., causes economic losses to tomatoes, peppers, cucurbits, lima, and snap bean crops^1^. The most effective control strategies of *Phytophthora* require a combination of different methods^1^. However, chemical control of *Phytophthora*, is becoming less effective because of growing resistance to existing chemicals, and because oomycetes lack the targets for most of the broad-spectrum agrochemicals deployed against phytopathogenic fungi. Since metabolism and nutrient acquisition play an important role in disease establishment, infection, and development of plant pathogens^2^ a complete, detailed analysis of metabolic strategies used by foliar oomycete pathogens is warranted to identify new strategies for control of these pathogens^3^. The biosynthesis of purine and pyrimidine nucleotides which are critical substrates for growth and replication, can proceed via *de novo* synthesis from amino acids, or by salvage pathways that enable the recycling of bases and nucleosides formed during the degradation of RNA and DNA^4^. While the enzymatic strategies for synthesis of purine and pyrimidine are strongly conserved across the domains of life, different salvage pathways are seen in mammals, plants, and fungi^5,6^

Earlier work indicated that during the initial biotrophic phase of the foliar infection by the potato blight pathogen *P. infestans*, biosynthesis of pyrimidines was strongly upregulated, indicating that invasive growth was not dependent on plant nucleotides, while the salvage pathway appeared to become more important during the necrotrophic stage of infection^7^. The nucleotide salvage pathway has also been reported to be upregulated in the switch from one development stage to another, such as in cyst germination^7^.

Uridine phosphorylase (UP) (E.C. 2.4.2.3), a key enzyme involved in the pyrimidine salvage pathway, in mammals and some bacteria, catalyzes the reversible phosphorolysis of uridine to uracil and ribose-1-phosphate^8^. Enzymes that catalyze the phosphorolytic cleavage of the glycoside are highly divergent in nature, but nonetheless share one of two protein folding patterns. Members of the NP-1 family include purine nucleoside phosphorylase (PNP, EC 2.4.2.1), 5′-deoxy-5′-methylthioadenosine phosphorylase (EC 2.4.2.28), uridine phosphorylase, and AMP nucleosidas^9^. NP-1 family encompasses enzymes that share a common single-domain subunit that display an α/β-fold with either a trimeric quaternary structure usually found in mammals, or a hexameric quaternary structure which is more typical in bacteria. The functional unit of NP-1 family is a dimer with the active site at the dimer interface, with amino acids in the active site contributed by each monomer^10^. The crystal structures of several members of the NP-I family have been determined, including purine nucleoside phosphorylase and uridine phosphorylase from human^11^, bovine^12^, and bacteria^9,13,14^. The chemical reaction of PNP, which has been extensively investigated by isotope effects, theoretical methods and inhibition is thought to proceed via an oxocarbenium ion intermediate^12,14–17^. The reactive-intermediates properties of carbenium ions are of particular interest, because they may also function as intermediates in the porphyrin biosynthesis^18^, sterol biosynthesis^19^ and the thiazole/pyrimidine coupling reaction involved in thiamin phosphate formation^20^. Using a comparative genomics approach, we identified two highly divergent genes as predicted uridine phosphorylases in *Phytophthora* genomes. Each of these genes were so divergent that a BLAST analysis using one gene identified the other sequence in the same genome, with an E value of only 0.2 and a sequence identity of 20%. Here we describe the crystal structure of PcUP1, and catalytic and biological properties of both genes.

We have solved the crystal structure of *P. capsici* uridine phosphorylase (PcUP1) in the native state, as well as bound to different ligands. Comparing to the typical trimeric or hexameric uridine phosphorylase, the PcUP1 display a different dimeric structure and there is no metal ion intermolecularly coordinated in the functional dimer. To determine the preferred activity of PcUP1, a series of pyrimidine bases or nucleosides were used for crystal soaking and crystallization experiments, and activity analysis. The result of the activity analysis support that both enzymes are functional uridine phosphorylases. Our structural analysis of key conserved residues surrounding the enzymatic pocket provides additional insight on the catalytic mechanism of UPs. This study describes two examples of widely divergent *UPs* in *Pythium* and *Phytophthora* genomes to optimally utilize nucleotide resources under different conditions. The high level of upregulation of *PcUP1* and *PcUP2* within 90 min after infection suggests that oomycete hyphae are already utilizing nucleotides that are exported from host tissues, a strategy that is not available for obligate oomycete pathogens, since these enzymes are not present in those genomes. Transcriptomic analysis of very early time points following infection of leaves with zoospores may lead to the identification of effectors mediating this process.

## Results

### Phylogenetic analysis of *UPs* in oomycete

**Figure 1:**
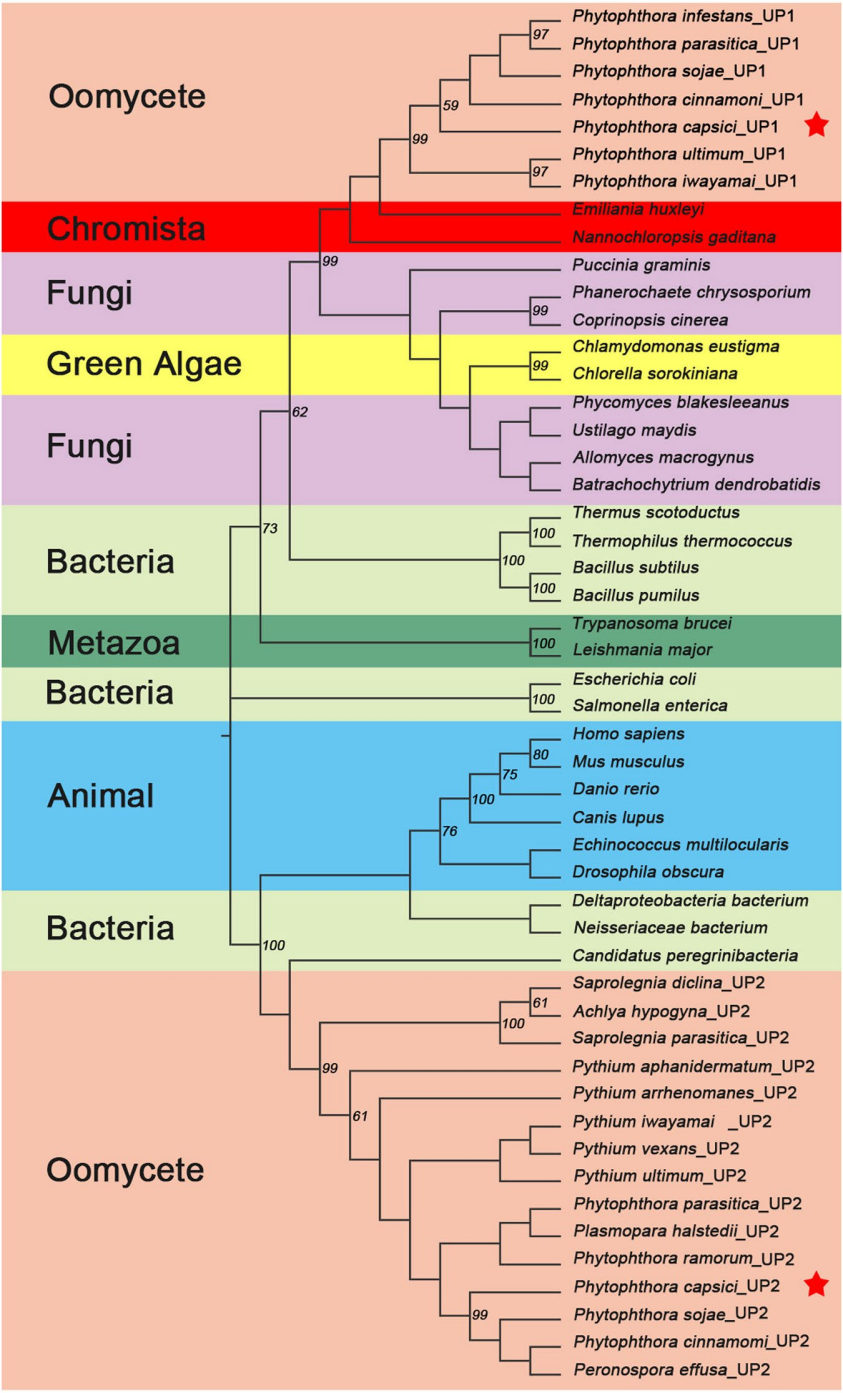
Phylogenetic analysis of *UPs*. PcUP1 and PcUP2 are marked by the red five-pointed stars.

The expression patterns for oomycete enzymes in the pyrimidine biosynthesis and salvage pathways were first described for the potato pathogen *P. infestans*^7^. BLAST analysis of the *P. capsici* orthologue *PcUP1* against other oomycetes revealed orthologues in all the other *Phytophthora* species as well as *Pythium* species. However, many of the gene models in *Pythium species* were incomplete due to sequencing gaps in the genomes, and have not been included in the alignment file used to generate a tree (Fig. 1). Notably, *UP1*-type genes were not present in any of the other phylogenetic groups of oomycete genomes in the FungiDB dataset. Thus *UP1*-like genes were absent from *Saprolegnia spp*., *Aphanomyces spp., Hyaloperonspora arabidopsidis*, *Bremia lactuca*, or *Albugo spp*. The closest gene models to *UP-1* sequences were those in single celled green algae (*Chlorella* and *Chlamydomonas spp*.), the haptophyte, *Emiliana huxleyi*, the stramenopile*, Nannochloropsis gaditana* and a few select fungal species. However, *UP1*-like genes are also not widespread in the fungal kingdom. In fungi, the primary pyrimidine salvage enzyme is trifunctional uridine nucleosidase/nicotinic acid riboside hydrolase *UrH1*^5^. Some, but not all of the fungal species that also have acquired *UP1* (Fig. 1) have also retained *UrH1*. Blast analysis of *UrH1* orthologs against oomycete genomes, also revealed the presence of this enzyme in oomycetes (Supplementary Fig. S1). *UrH1* orthologs were detected in *Aphanomyces*, *Phytophthora*, and *Pythium and Saproglegnia spp*., as well as the obligate pathogen *Bremia lactuca*.

*PcUP1* is highly divergent from previously characterized, and crystalized UPs, so we used other known gene models to query the *P. capsici* genome for additional gene models. BLAST analysis of *HsUPP1*, against the *P. capsici* genome revealed a second genome model, *PcUP2*, which was so divergent from *PcUP1*, that when *PcUP2* was used in a BLAST search of the oomycete genome, the other gene sequence had only an E value of 0.2 and a sequence identity of 20%. Gene models of this type were also present in *Saprolegnia, Aphanomyces, Pythium and Phytophthora spp*., but absent from the sequenced genomes of four obligate oomycete pathogens. Thus, these obligate pathogens do not have a complete pyrimidine salvage pathway. However, the downy mildew pathogen of sunflowers *Peronsopora effuga* has a *UP2*-like gene. Oomycete *UP2*-like gene models cluster strongly with *UP* genes in metazoans (Fig. 1). To gain a better insight on the roles of these divergent *UP* proteins, we crystalized PcUP1 and examined PcUP1 and PcUP2 catalytic activity and expression at various developmental stages. These results are described below.

### Overall structure of *P. capsici* uridine phosphorylase1

**Figure 2:**
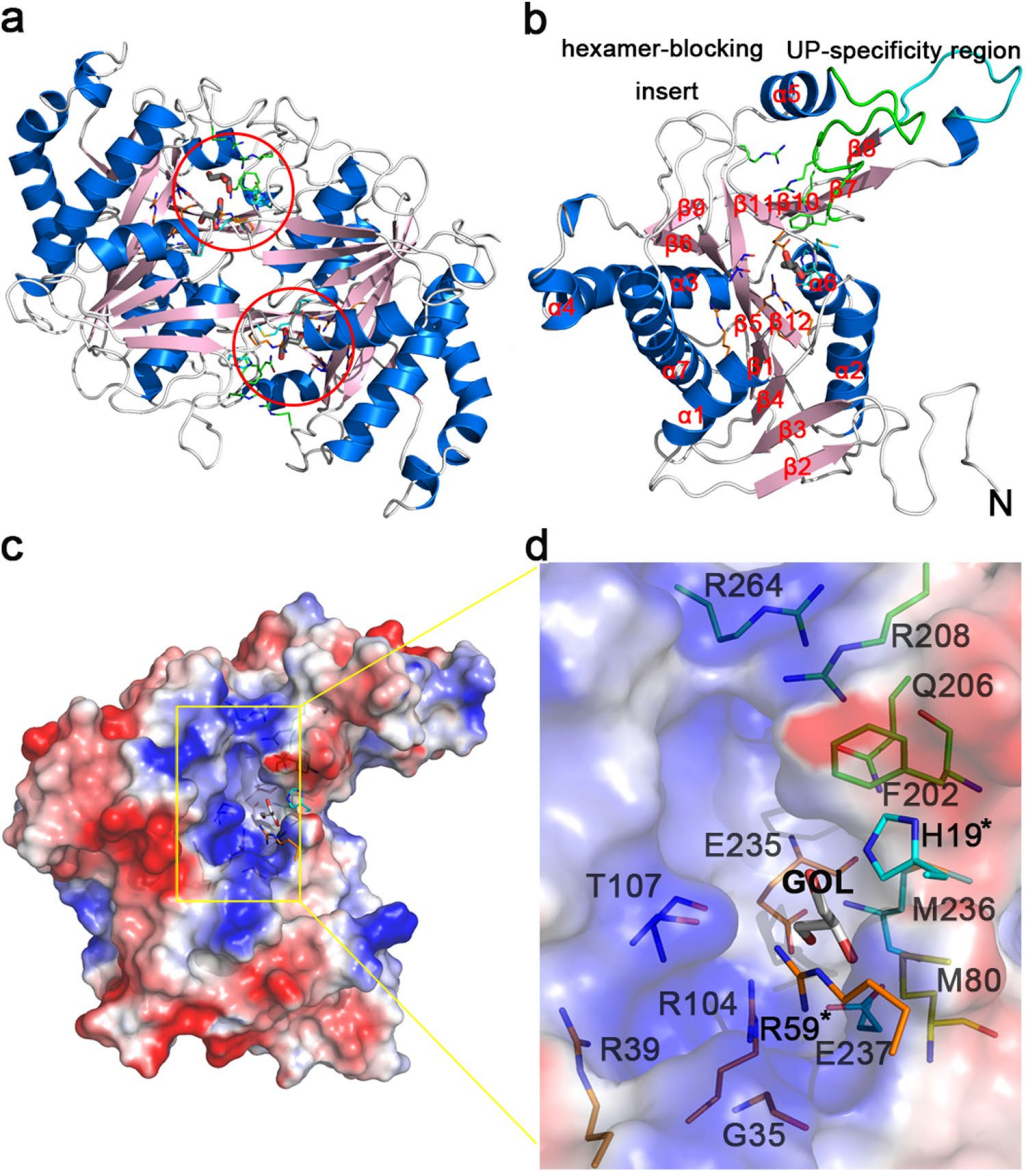
Overall structure of PcUP1. (**a**) PcUP1 Dimer. The dimer is presented as a cartoon model with α-helices colored marine, β-strands colored light pink and loops colored white. The active pocket is marked in a red circle.In the circle, glycerol is shown as stick and active residues of the interaction network is shown as lines. (**b**) PcUP1 monomer. The monomer is presented as a cartoon model with α-helices colored marine, β-strands colored light pink and loops colored white with the UP-specificity region green and the hexamer-blocking inserts cyan. (**c**) Electrostatic surface presentation of native PcUP1 monomer structure. Glycerol is shown as stick and active residues of the interaction network is shown as lines. (**d**) The larger features are also labeled on the right. His19 and Arg59, marked with an asterisk, are contributed by the neighboring monomer.

**Figure 3:**
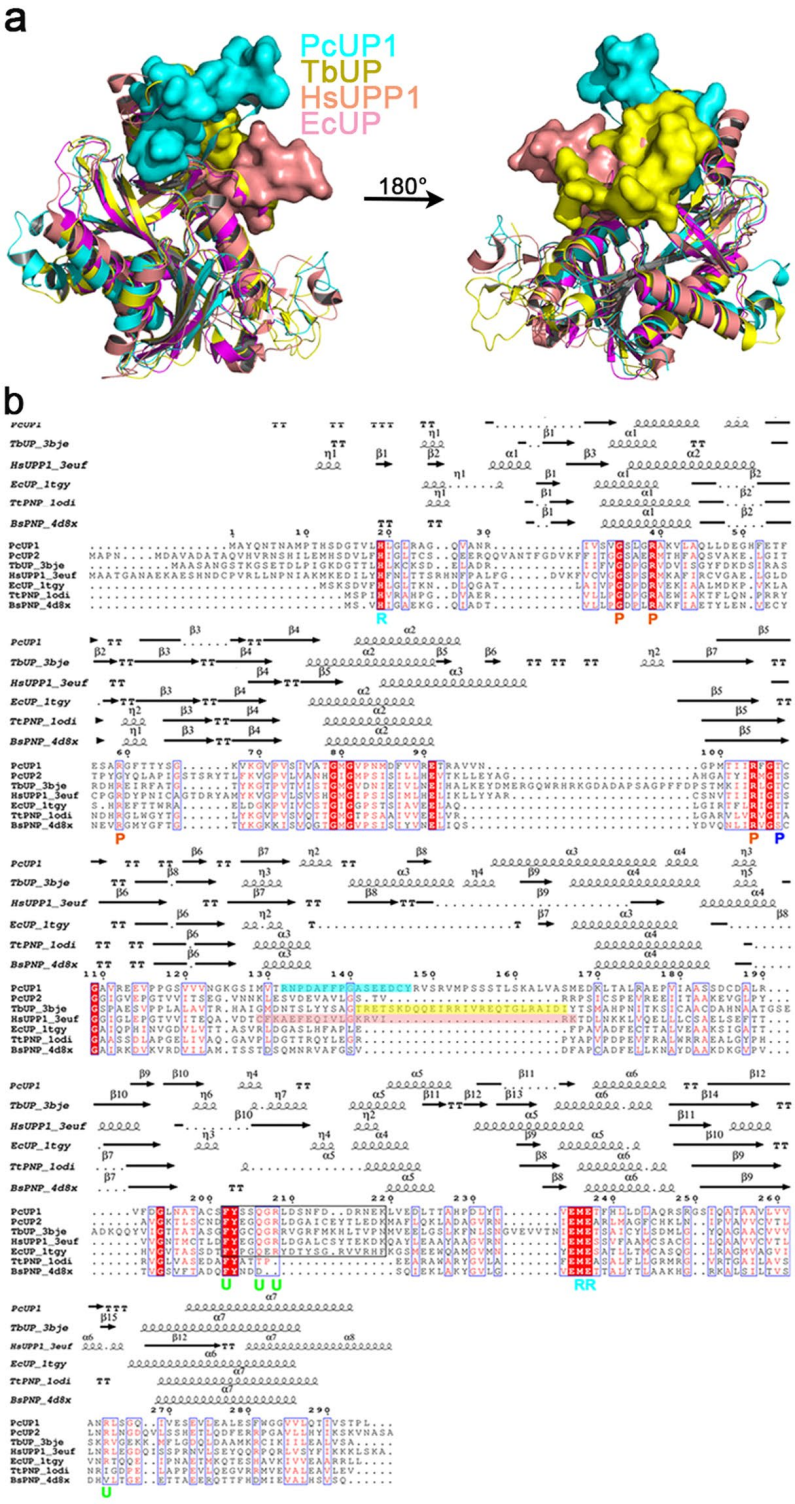
Structural comparation and sequence alignment between uridine phosphorylases and purine nucleoside phosphorylases. (**a**) Comparation of structures among uridine phosphorylases. The hexamer-blocking insertions are shown in surface. PcUP1 in cyan; TbUP (PDB: 3bje), *T. brucei* uridine phosphorylase in yellow; HsUUP1 (PDB: 3euf), *H. sapiens* uridine phosphorylase1 in salmon; EcUP (PDB: 1tgy), *E. coli* uridine phosphorylase in magenta. **(b**) Structure-based sequence alignment of representative uridine phosphorylases together with purine nucleoside phosphorylases. Amino acid numbering and secondary structural elements of PcUP1 TBUP; HsUUP1; EcUP; TtPNP (PDB: 1odi), *T. Thermophilus* purine nucleoside phosphorylase; BsPNP (PDB: 4d8x), *B. subtilis* purine nucleoside phosphorylase are mapped at the top of the alignment. UP-specific inserts that block hexamer formation of PcUP1, TbUP and HsUUP1 are shaded cyan, yellow and salmon separately. The UP-specificity region is marked by the black box. The key substrate/product interacting residues are labeled “U” for uracil, “R” for ribose, “P” for phosphate. Structure-based sequence alignment was created using Clustal X Version 2.0^54^ and ESPript 3.0^55^.

The recombinant protein of wild-type PcUP1 was expressed and crystallized in the orthorhombic space group *P2*_*1*_. The structure of PcUP1 was refined to 1.57 Å resolution. Two polypeptide chains formed an asymmetric homodimer and each monomer contains one active site (Fig. 2a). Each monomer of PcUP1 consists of a central twelve-stranded mixed β-sheet surrounded by seven α-helices (Fig. 2b). Although the homodimer’s conformation of PcUP1 is equivalent to the dimer unit in the typical NP-I subfamily, it is not possible to assemble three PcUP1 dimers into the canonical hexamer as a result of a 16-amino-acid insertion in the sequence of PcUP1 (Figs. 2b and 3b). This *P. capsici*-specific insert, creates an additional secondary structural element, that protrudes into the space that would be occupied by the neighboring dimer of the canonical NP-1 hexamer (Supplementary Fig. S2a–c) thus sterically blocking trimerization of the dimers (Fig. 3a). TbUP and HsUPP1 also harbor hexamer-blocking insertions (Fig. 3a,b). The strictly ear-shaped conserved catalytic pocket with positive charge of PcUP1 is located on the monomer-monomer hydrophobic interface (Fig. 2c,d). As predicted from sequence alignment with homologous enzymes, although the sequences homology is not high, the three dimensional structures of uridine phosphorylases and purine phosphorylases from various species are highly conserved (Fig. 3b).

### Intermolecular metal-binding site

**Figure 4:**
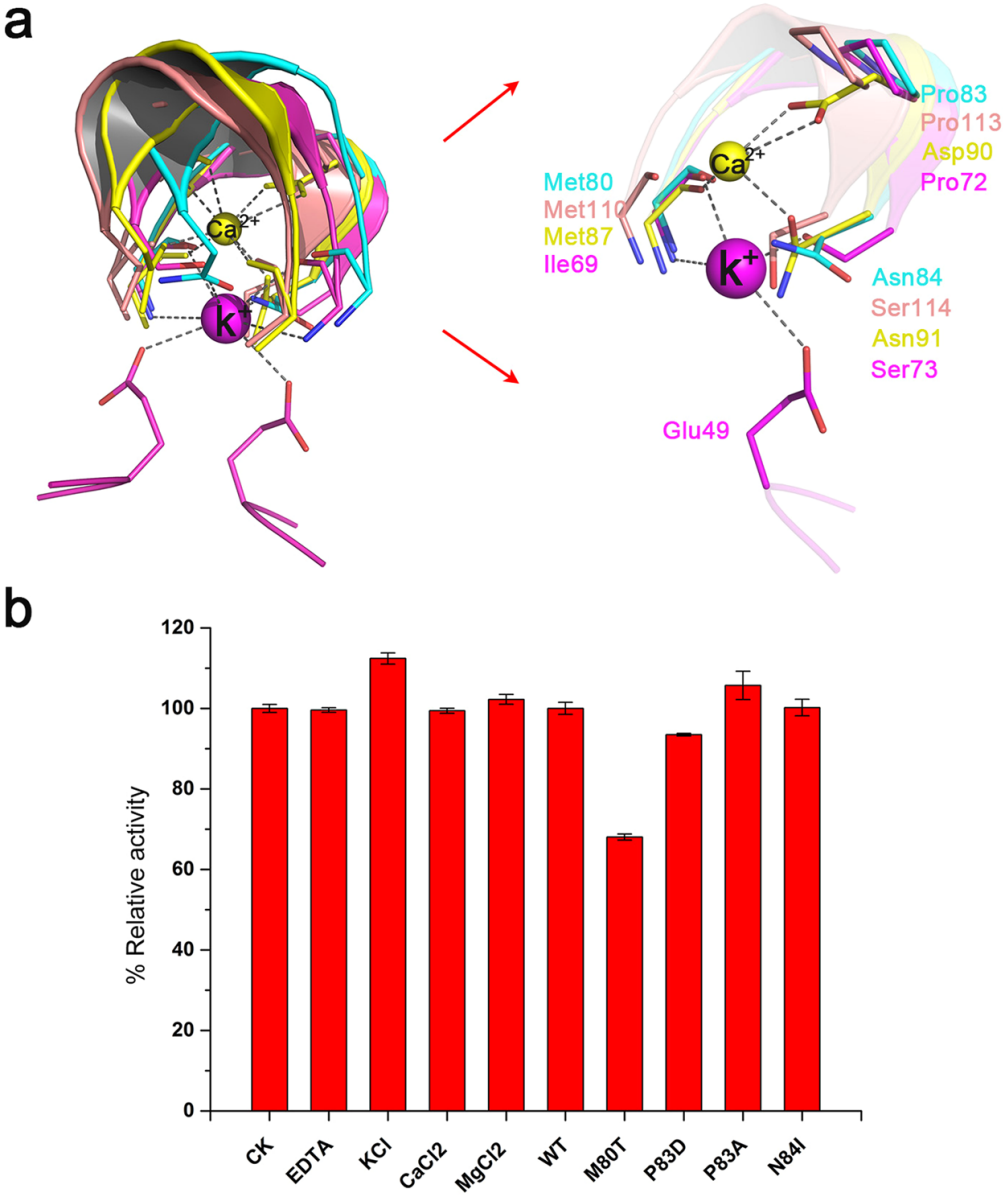
Metal ion-interacting network of UPs. (**a**) Superimposition of the metal ion-interacting network of PcUP1 with other UPs. PcUP1 is colored cyan; TbUP is colored yellow; EcUP is colored magenta; HsUPP1 is colored salmon. The shown residues are strictly conserved or semi-conserved. (**b**) Effects of different metal ions and mutational protein on PcUP1 activity. The production levels of URA by each protein are presented as percentages of the wild type. Each measurement was conducted in triplicate, from which the average ± s.d. was calculated.

Uridine phosphorylases can be divided into two categories based on the presence or absence of metal ions. *E. coli*^21^ and *Salmonella typhimurium* enzymes^22^ contain a potassium ion which is situated between active sites in the dimer interface. In the protozoan pathogen, *Trypanosoma brucei*, a calcium ion fulfills this role^10^. Each monomer provides three amino acids to coordinate with the metal ion. Enzymes that contain no metal ions, include *Homo sapiens* and *Schistosoma mansoni*^23^ and PcUP1 belongs to the latter category (Fig. 4a). Comparative analysis of the metal binding pocket of homologous enzymes reveals conservative amino acids around the metal-binding network (Fig. 4a). In TbUP a triad of Met81-Asn90-Asp91 coordinately binds a calcium ion, while in PcUP1 the equivalent residues are Met80-Pro83-Asn84.

While PcUP1 does not contain a metal ion, the conservation of amino acid residues surrounding this pocket suggests that they might be critical for catalysis. However, the catalytic activity of variants Pro83Asp and Pro83Ala is similar to that of the WT (Fig. 4b). Among the four mutants (Met80Thr, Pro83Asp, Pro83Ala, Asn84Ile) in the intermolecular mental-binding pocket, only the mutation of Met80Thr results in enzyme inhibition. The lower activity of the mutant may be caused by the collapse of the catalytic channel, as Met80 is near the pocket (Fig. 2d). The addition of either EDTA or calcium magnesium, or potassium ions has no effect on enzyme activity (Fig. 4b). In UP enzymes with calcium or potassium ions in the pocket, the metal ions play a structural role enabling tighter binding of the dimer complex^10^. The buried area of dimer interface of PcUP1 and other enzymes that form dimers is much larger than that of hexameric quaternary structure (Supplementary Fig. S2d). Perhaps, PcUP stabilizes its homodimeric structure via an alternative strategy, reconfiguring the structural elements of the would-be hexamer interface to increase the size of the dimer interface, so that they interact with its monomeric partner instead of another dimer.

### Active site pockets and bound ligands

In the native structure of PcUP1, glycerol which was included as a cryoprotectant, was present in the active site pocket of each monomer (Fig. 2c,d). Three crystal structures of PcUP1 in complex with uracil/ribose-1-phosphate (2.5 Å  resolution), 2′-deoxyuridine/phosphate (2.1 Å resolution) and thymidine/phosphate (1.97 Å resolution) were solved. In the uridine/phosphate UP complex, the products of the catalytic reaction, ribose-1-phosphate and free uracil were present, judging from the strong difference density peaks of definable shape in the active pocket of each monomer (Supplementary Fig. S3a), while in the 2′-deoxyuridine/phosphate UP complex, the continuous electron density for the complete substrate was present in the active sites (Supplementary Fig. S3b). In the thymidine/phosphate UP complex, the active pocket in one of the monomers was occupied by the substrate (Supplementary Fig. S3c). Comparison of the ligand binding in each complex, showed that residues forming the cavity, adjusted slightly to accommodate different substrates (Supplementary Fig. S3d–f).

**Figure 5:**
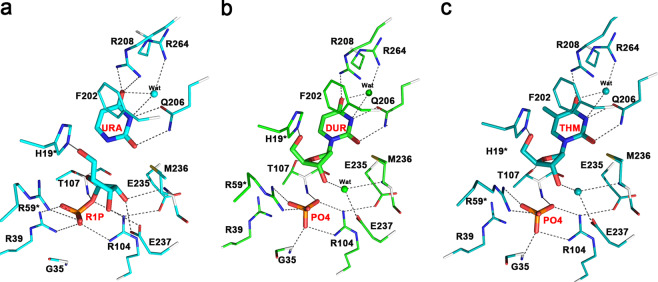
The active site of PcUP1 complexed with substrates. Substrate and product-binding residues. (**a**) Enzyme–ligand interaction networks of Uracil with ribose-1-phosphate, (**b**) 2′-Deoxyuridine with phosphate and (**c**) Thymidine with phosphate are shown in stereo view. The ligands and protein residues are presented as thick and thin sticks, respectively. Distances <3.5 Å are indicated with dash lines.

The amino acids that compose the active site pocket are derived from one of the monomers, but two that make crucial interactions with ligands, namely His19 and Arg59, are contributed by the neighboring monomer (Fig. 5a–c). The ribose moiety, either as the ribose moiety of a nucleoside or that of the ribose-1-phosphate is bonded by the side chain His19 and Thr107 with the 5′hydroxyl and O4 atom in the form of hydrogen bonds, respectively (Fig. 5a–c). The hydrogen bonds can also be formed by M236 and E237 with the 2′hydroxyl and 3′hydroxyl of the ribose-1-phosphate (Fig. 5a), while in the ribose moiety of a nucleoside, the bonds are formed via an active water (Fig. 5b,c). Arg59 is an important residue in the phosphate-bonding pocket, forming two hydrogen bonds with the free phosphate and one with the phosphate moiety of ribose-1-phosphate. The phosphate is further bound by the main chain nitrogen of Gly35 and Thr107 and the side chains of Arg39, Arg104. Notably, the Arg39 only bonds with the phosphate group of R1P, but not the free phosphate, which is inconsistent with the previous description of the bacterial enzyme^21^. And the Arg104 is further stabilized by Glu235 and Thr238 (Fig. 5a–c). In fact, Gln206 and Arg208 that interact with the uracil or uracil-analogue, have no corresponding amino acids in PNPs. Thus, the presence of these residues can be used to differentiate UP and PNP enzymes^10^.

Superimposition of the native and complex structures shows that substrate binding causes many active residues of complex structures move to form a tighter pocket (Supplementary Fig. S4). The conformational changes of His19, Arg39, Arg59 and Thr107 are especially notable. The Arg39 residue of the product complex turns more than 45 degrees compared to the residue of native structure, and the hydroxy group of Thr107, influenced by O_4_ atom of the ribose moiety, rotates around the main chain (Supplementary Fig. S4). In contrast, other active residues like Met236 and Glu237 change slightly. A small conformational change may be because the native PcUP1 active site is not entirely devoid of ligands. A molecular water is observed in the ribose moiety binding region of the substrate complex, but not in the product complex (Fig. 5a–c). Met236 and Glu237 are farther away from the ribose moiety of the nucleoside than from the moiety of ribose-1-phosphate, so that a water is needed when Met236 and Glu237 stabilizing the former ribose moiety (Supplementary Fig. S4b).

### Site directed mutagenesis of PcUP1 and PcUP2

**Figure 6:**
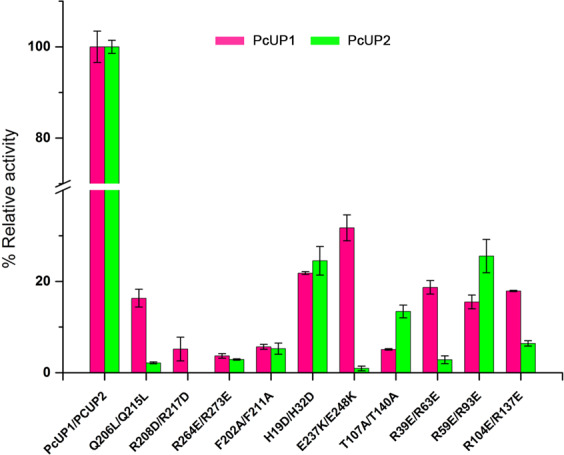
Relative activity of site-directed mutants of PcUP1 and PcUP2. Enzyme activity rates were compared to those of the native protein. Individual mutations reduced uridine phosphorylase activity by more than 50%. Mutation of residues that interact with the uracil or uracil-analogues exhibited lower enzyme activity than other mutants. Each assay was conducted in triplicate, from which the average ± s.d. was calculated.

To further characterize the importance of amino acid residues surrounding the substrate binding pocket, we constructed and measured the catalytic activity of several mutants of PcUP1 and PcUP2 (Fig. 6). Key residues of purine nucleotide phosphorylases have previously been characterized by alteration of key residues to neutral amino acids^4,14^. Here we used site-directed mutagenesis to alter the charge of individual amino acids surrounding the catalytic pocket. Variants of the key substrate discriminating residues Gln206Leu/Gln215Leu and Arg208Asp/Arg217Asp in the UP-specificity region exhibit very low activity, suggesting that the two amino acids are essential. Mutation of Arg264Glu or Arg273Glu also resulted in an almost complete loss of enzyme activity, indicating the critical role of the positively charged arginine for substrate binding. A phenyalanine residue is strongly conserved in UP*s* (Fig. 3b) and mutagenesis of this residue Phe202Ala/Phe211Ala resulted in an almost complete loss of activity. The relative activity of Glu237Lys in PcUP1 (32%) is relatively higher than that of Glu248Lys (1%) in PcUP2. Thr107 in PcUP1 and Thr140 in PcUP2, which interacts with ribose and phosphate, is also essential for PcUPs catalysis activity as Thr107Ala exhibited 5.08 ± 0.16% URA production and Thr140Ala exhibited 13.4 ± 1.39% URA production. In addition, the primary phosphate-interacting residues including Arg39/Arg63, Arg59/Arg93 and Arg104/Arg137 are also essential for the catalytic activity, as each corresponding Glu variant showed lower activity.

### Conformation of substrates in complex with uridine phosphorylase

In the pyrimidine molecule, the glycosidic bond can be rotated and the rotation angle is defined by the glycosidic bond torsion angle O4′-C1′-N1-C2 (according to the IUPAC nomenclature) denoted χ. Ranges of 0–90° and 270–360° characterize for syn, and 90–270° for anti^24^. The torsion angle of uridine in complex with uridine phosphorylase are 134° while in the complex of PcUP1 the angle of DUR and THM are 179.75° and 173.92° (Supplementary Fig. S3b,c). According to the potential energy curves of the ground state of the uridine^25^, the relative energy of DUR and THM is much lower than that of uridine, so the torsion stress on the glycosidic bond facilitates catalysis.

### Expression analysis of *PcUP1* and *PcUP2* as a function of development

**Figure 7:**
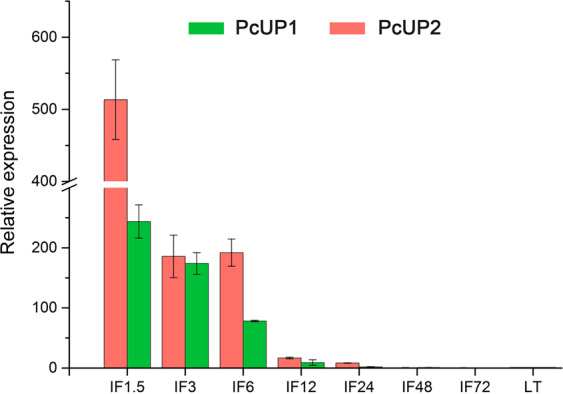
Relative expression of PcUP2 and PcUP2 after (IF) of pepper leaves, respectively. Expression levels were calculated relative to levels in mycelia. The data represent the mean ± SD from at least three independent experiments.

As previously noted, a pyrimidine salvage pathway is present in most organisms. In Fig. 7 the expression of *PcUP1* and *PcUP2* in infection stages at 1.5, 3.0, 6.0, 12.0, 24.0, 48 and 72 h (hpi) is shown relative to their expression level in mycelia grown on oatmeal agar. At 1.5 h after infection on leaves, *PcUP1* is upregulated 250 fold while *PcUP2* is upregulated more than 500 fold. At this early stage of the biotropic infection, formation of haustoria in *P. sojae* has already started^26^. Thus, the elevated expression of both genes suggest that the pathogen may already be acquiring substantial amounts of nucleotides from the host.

### The enzymatic assays

To characterize the enzymatic activity of two proteins, we determined Michaelis-Menten kinetics for each enzyme. The K_M_ and K_cat_ for UP1 was estimated at 6.96 ± 1.52 mM and 982± 152 sec ^−1^. The K_M_ and K_cat_ for UP2 was 7.55 ± 0.6 mM and 1188 ± 90 sec^−1^ respectively, with the number of substrate sites (**Et)** per active molecule listed as two Fig S5. Thus despite having a low level of sequence identity, both of these enzymes share similar kinetic properties. Notably the affinity of both enzymes for uridine is much less than that those of previously characterized enzymes ^14,27^, but K_cat_ values are also higher. The *E. coli* enzyme did not exhibit cooperative binding of substatrates^21^, although hUP did exhibit positive cooperativity of substrate binding^27^. Kinetic analysis of UP1 and UP2 is not consistent with a strong effect of substrate cooperativity (Supplementary Fig. S5).

### Possible mechanism of catalysis

Two main mechanistic models have been proposed for transition state stabilization in PNP. One model comprises an electron-rich purine produced by a flow of electrons from the O4′ of the ribose moiety to the purine^4,28^. The second model contains protonation at N7 of the purine to generate an electron-deficient purine, thereby allowing withdrawal of electrons from the scissile bond^29^. Due to the similarity of structures between UP and the NP-I phosphorylase, and the highly conservative phosphate and ribose binding pockets (Fig. 3), it is reasonable to conclude that UP may have a similar catalytic mechanism to PNP^21^.

We suggest that PcUP catalyzes the reversible phosphorolysis of uridine to uracil and ribose-1-phosphate using the similar substrate-assisted catalytic pathway proposed in PNP from human erythrocytes based on the structural data and site-directed mutagenesis of PcUP1 and PcUP2. In Purine Nucleoside Phosphorylase of human erythrocytes, formation of a high-energy ground state complex and stabilization of the transition state are the two significant constituent parts in the efficient catalysis. In the direction of phosphorylation, glycosidic bond cleavage is promoted by binding a high-energy nucleoside conformation and by producing a electrostatic strain. Electrostatic strain is invoked by the phosphate deprotonation, which is caused by a positively charged histidine. The deprotonation increases phosphate nucleophilicity and puts greater negative charge on the endocyclic ribosyl oxygen. The increasing negative charge on the phosphate oxygen leads to repulsion between the electrons of phosphate oxygen and electrons on the endocyclic ribosyl oxygen, which results in electrostatic strain and weakening of the glycosidic bond. As glycosidic bond breakage proceeds, an intermediate oxocarbenium ion, stabilized by the phosphate dianion, is formed, which is the key structural feature that differentiates phosphorylases from nucleosidases (e.g., AMP nucleosidase) and glycosidases (e.g., lysozyme). Glycosidic bond cleavage leads to a electron density accumulation of the purine ring, the resulting negatively charged purine base is stabilized by a hydrogen bond formed between an Asparagine and N7. This hydrogen bond preferentially stabilizes the transition state-intermediate oxocarbenium ion. Therefore, the cleavage of glycosidic bond proceeds through a TS that entails a “push-pull” type effect in which the ionization of phosphate pushes electrons from the endocyclic ribosyl oxygen into the C1′-O bond, thereby weakening the glycosidic bond, while at the same time the Asn243-N7 hydrogen bond pulls electrons from the glycosidic bond onto the purine ring. After glycosidic bond is broken, ribose-1-phosphate dissociates from the active site and the negatively charged purine is reprotonated by water. The final step is release of the neutral purine base.

However, several unique features are present near the catalytic center. First, in the phosphorolytic direction, it is Arg104 that causes the phosphate monoanion deprotonation, not a His residue as is the case in human PNP. The positively charged Arg is stabilized by two amino acids Glu235 and Thr238, forming a catalytic triad. In addition to the charge-transfer relay system, the reorientation of Arg39 pushes the phosphate closer to the ribose moiety. As a result, the negative charge of the endocyclic ribosyl oxygen is strengthened by the nucleophilic phosphate dianion. As negative charge on the phosphate oxygen increases, electrostatic strain is generated by the electron repulsion between electron pairs on the endocyclic ribosyl oxygen and the negatively charged phosphate oxygen, thus leading to weakening of the glycosidic bond. As glycosidic bond cleavage proceeds, an intermediate oxocarbenium ion comes into being and stabilized by the phosphate dianion concurrently. Second, glycosidic bond breakage results in electron density accumulating on the pyrimidine ring. The resulting negatively charged pyrimidine base is stabilized by a hydrogen bond formed between Gln206 and N1, while in HsPNP it is the Asn residue bonding with the N7. Arg208 and Arg264, the latter via a water molecule, also contributes to the stabilization of the pyrimidine ring.

**Figure 8:**
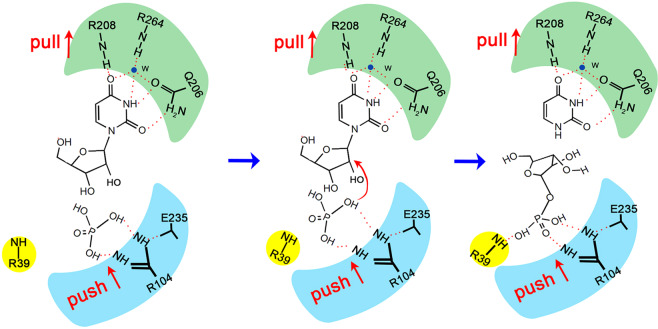
Proposed catalytic mechanism of PcUP1. In this model, deprotonation of phosphate by Arg104, increases the negative charge on the phosphate oxygen, leading to repulsion between the electrons of phosphate oxygen and electron pairs of the endocylic ribosyl oxygen and the formation of an intermediate oxocarbenium ion. At the same time, the Gln206, Arg208 and Arg264-N1 hydrogen network-bond pulls electrons from the glycosidic bond onto the pyrimidine ring. These two actions work in concert to weaken the glycosidic bond. Arg39 (shown in yellow) pivots, to physically push the phosphate closer to the ribose moiety. After glycosidic bond cleavage, ribose-1-phosphate dissociates from the active pocket, and then an active water reprotonates the negatively charged purine. The final step is to release the neutral purine base.

To summarize, cleavage of the glycosidic bond processes through a “push-pull” type effect (Fig. 8) as described earlier for HsUPP1^30^. In this model, deprotonation of phosphate by Arg104, increases the negative charge on the phosphate oxygen, leading to repulsion between the electrons of phosphate oxygen and electrons on the endocyclic ribosyl oxygen. At the same time, the Gln206, Arg208 and Arg264-N1 hydrogen network-bond pulls electrons from the glycosidic bond onto the pyrimidine ring. These two actions work in concert to weaken the glycosidic bond. Arg39 pivots to physically push the phosphate closer to the ribose moiety. After glycosidic bond cleavage, ribose-1-phosphate dissociates from the active pocket, and then an active water reprotonates the negatively charged purine. The final step is to release the neutral purine base.

## Discussion

The pyrimidine salvage pathway is a core metabolic strategy for most organisms. What is particularly notable about pyrimidine salvage in *Phytophthora* and *Pythium spp*. is that these genomes contain two distinct, but kinetically similar Ups, in addition to a UrH1-like enzyme. In contrast to UP enzymes, UrH1 enzymes are unidirectional, and function to convert uridine to uracil and ribose. The spike in the expression of both PcUP enzymes during infection is both large and very short, with peak expression occurring within 1.5 h after inoculation of leaf tissue. As more biomass of the oomycete pathogen is synthesized in the leaf tissues, the relative expression rates drops precipitously, but at 6 h, the expression of *PcUP1* and *PcUP2* is still 70 fold and almost 200 fold respectively above levels observed in rapidly growing mycelia on media. The virulence and metabolic strategies of oomycete pathogens is still at a very early stage of investigation^3^ and it is possible that the short-term upregulation of *UP* genes is part of a particular virulence strategy of the pathogen that is activated immediately after infection.

Cluster analysis of the transcriptomic analysis of *P. palmivora* infection of *N. benthamiana* show that the transcriptomic profile at 6 h is distinct from that at 12 h and 18 h, but this entire time period has been traditionally grouped as the biotrophic phase of *Phytophthora* infection^31^. Orthologs of a haustoria-specific membrane protein peaked between 3 h and 6 h and then decreased afterward. A detailed cytological analysis at very early time periods after infection has not been made for *P. capsici* and pepper. However, in compatible interactions of *P. sojae* on soybean hypocotyls, hyphae had penetrated through the first layer of the cortex of soybean hypocotyls by 2 h and had reached the third layer by 3 h^26^. In infection of soybean roots, wall appositions were very prevalent with 30 min^32^. Based on the hypothesis that host amino acids could represent an important nutrient source for invading pathogens, it has been hypothesized that some pathogen effectors might modulate the activity of plant transporters to increase the export of amino acids into the apoplast^33^. Consistent with this hypothesis, mutations in the gene RPTs1 resulted in resistance to *P. parasitica* and two other biotropic pathogens *Pseudomonas syringae pv. tomato* (Pst) DC3000, *Golovinomyces cichoracearum*^34^.In a similar vein, we hypothesize that *P. capsici* may modulate host transporters to enhance export of nucleotides. Enhanced import of host nucleotides by hyphae at very early stages of infection, would necessitate high levels of expression of nucleotide salvage enzymes. It is also critical for the pathogen, that the plant tissues do not respond defensively to the export of purine or pyrimidine nucleotides. For example, elevated levels of ATP in the apoplast are sensed by the membrane receptor DORN1 protein which functions as an extracellular signaler of ATP^35^. When DORN1 perceives higher levels of external ATP, it activates the expression of many defense response genes^36^. Moreover, constitutive expression of this gene in *Nicotiana benthamiana* enhances resistance to *P. capsici*^37^. On the pathogen side, the *ipiO* effector gene from *P. infestans* is expressed early in infection^38^ and is capable of targeting this receptor, also named as LecRK1.9 to suppress plant responses, and thus confer increased susceptibility to biotrophic pathogens^39^. Thus, effectors may be required to enhance the export of purine and pyrimidine nucleotides levels to the apoplast, and also to silence defense responses that are activated in response to this metabolic switch. Transcriptomic profiling of very early time periods following infection may be useful in identifying effectors that modulate host metabolism and transport.

*UP2* is found in *Saprolegnia* and *Aphanomyces spp*., basal members of the oomycete tree as well, as *Phytophthora* and *Pythium* species (Fig. 1). Therefore, *UP2* was likely present in the ancestral oomycete progenitor. In *Saprolegnia spp*. there are two *UP2* genes which arose from a duplication event. *Pythium* and *Phytophthora* genes have two unrelated *UP* genes and have thus utilized a different evolutionary strategy from that of fish pathogens which arose from a tandem duplication event. *UP2* genes cluster with similar genes from some fungal species. These data suggest a horizontal transfer event, although the source of that cannot be predicted from these data. Horizontal transfer events have profoundly altered metabolic biosynthetic pathways in oomycetes, relative other eukaryotic groups^40^. Thus far, 48 gene families which contribute to saprotrophic or necrotrophic phase of growth of oomycete pathogens have been described^41^. In contrast, obligate oomycete pathogens have lost specific metabolic pathways. These pathways include nitrate uptake and assimilation^42^ and here, we noted the absence of a pyrimidine salvage pathway in *H. arabidopsidis* and *Albugo spp*., although the lettuce pathogen *B. lactuca* has a *UrH1* gene (Supplementary Fig. S1). The loss of a key enzyme in the pyrimidine salvage pathway in obligate oomycete pathogens, suggests that they have a fundamentally different nutrient acquisition strategy than that of *Phytophthora* and *Pythium* species during the early stages of infection.

It may seem incongruous for *Pythium* and *Phytophthora spp*. to have acquired and retained two kinetically similar UPs, in addition to a *UrH1-*like enzyme for pyrimidine salvage. However, it cannot be assumed that both *UP* genes in plant pathogens or the duplicated genes of *Saprolegnia spp*. are localized to the same regions within the hyphae or appressoria. Notably, *PcUP1* and *PcUP2* are expressed at different rates during the very early stages of infection. Since *Phytophthora*, and *Pythium* genomes, also have a uridine hydrolase which catalyses uridine into uracil and ribose, there are three key enzymes in pyrimidine salvage pathways that can be spatially and temporally expressed during infection. These oomycete pathogens may share a nucleotide acquisition strategy during infection with the fungal pathogens that also have both a *UP* and a *UrH1* enzyme (Fig. 1, Fig. S1).

In this paper, we have structurally characterized a uridine phosphorylase from *P. capsici*, which has low sequence homology to *UPs* (Fig. 3b) not from other oomycetes. However, the three-dimensional structures among these enzymes is highly conserved (Supplementary Fig. S2a–c). There was no significant influence on the enzyme activity, when we mutated any of the putative metal ion-binding amino acids, and also added different metal ions in the catalytic reaction assay of PcUP1. This indicates that PcUP1 doesn’t require metal ions to stabilize its conformation.

Among the eleven mutations of PcUP1 and PcUP2, the degree of reduction in the enzyme activities was essentially the same except for the Glu237Lys/Glu248Lys mutants. The relative activity of Glu237Lys in PcUP1 (32%) is relatively higher than that of Glu248Lys (1%) in PcUP2. The different impact on the enzyme activity may be caused by the different degrees of collapse on the active pocket. In PcUP1, the three amino acids following Glu237 are Thr, Phe and His, while in PcUP2, they are Ala, Arg and Leu that follow Glu248. Perhaps the benzene ring of Phe and the imidazole ring of His are better able to support the active pocket. The crystal structure of PcUP1 shows that Phe202 is positioned to form a π-π stacking interaction with the nucleotide ring. The conservation of this residue in other UPs and the losing of catalytic activity resulting from the Phe202Ala/Phe211Ala mutation indicates the essential role for this residue. We hypothesize that Phe202 or Phe211Ala stabilizes the orientation of the pyrimidine ring to facilitate catalysis.

The analysis of three complex structures of PcUP1, including the structures of one product and two substrate analogues, together with a detailed site-directed mutagenesis of amino acid residues surrounding the substrate binding pocket also contributes to the understanding of the catalytic mechanism of these enzymes. Despite the variance of primary sequences, all the uridine phosphorylases, either PcUPs reported here or from other species, use a similar “push-pull” enzymatic strategy to catalyze the hydrolysis process.

## Materials and Methods

### *P. capsici* strain culture

*Phytophthora capsici* LT1534 strain was cultured on oatmeal agar at 25 °C. Zoospores were prepared for infiltration into leaves as previously described^43^.

### Sequence bioinformatics and expression analysis by qRT-PCR

BLASTp was used to retrieve the sequence of *PcUP1* using the *Phytophthora infestans* gene as a query sequence. The *PcUP1* sequence was then used to search for orthologs in oomycetes using Fungidb and to identify closely related genes in other genomes using the NCBI Database. *PcUP2* was retrieved by using a similar search of databases, starting with *HsUPP1* as a query sequence. These amino acid sequences were aligned by ClustalX^44^. The evolutionary tree was constructed using MEGA version 6.0^45^. Boot strapping support numbers that above 60% are shown at each node. Bar = 20% divergence.

Total RNA of *P. capsici* hyphae and total RNA of infected pepper leaves were isolated using Fungi RNA Kit and Plant RNA Kit (OMEGA), respectively following the manufacturer’s protocol. Integrity of RNA was assessed by agarose gel electrophoresis. The RNA was quantified using a spectrophotometer (IMPLEN NANOPHOTOMETER P-330-31). First strand cDNA was synthesized using HiScript II 1st Strand cDNA Synthesis Kit (VAZYME) using the kit protocol. Real-time qRT-PCR reactions using the prime sets described in (Supplementary Table S2) were performed using Cham SYBR color qPCR master mix kit (VAZYME) and run on a QuantStudio 6 Flex (LIFE TECHNOLOGY, USA). Ubiquitin c expression (UBC) was used as an internal control. Relative gene expression level was calculated using 2^−△△CT^ method.

### Gene cloning and protein purification

The *PcUP1* and *PcUP2* from *Phytophthora capsici* (GenBank accession nos. **MH244427** and **MN165113**) were cloned from genomic cDNA of *P. capsici* strain LT1534, ligated into the pET28a vector that includes a N-terminal hexahistidine tag using the primer sets described in (Supplementary Table S2), and expressed in *Escherichia coli* BL21trxB (DE3). Bacterial cultures were grown in LB medium at 37 °C until the OD_600_ reached 0.8 and then induced by addition of 1 mM isopropyl β-D-thiogalactopyranoside (IPTG) at 16 °C for 24 h. Cells were harvested by centrifugation at 6000 × g for 5 min, and then resuspended in extraction buffer containing 20 mM Tris-HCL, PH 7.5, 150 mM NaCl and 30 mM imidazole. The cells were lysed by a Ultrasonic Homogenizer (NINGBO SCIENTZ BIOTECHNOLOGY Co, Ltd) and the debris was removed by centrifugation (14000xg, 25 min 4 °C). The supernatant was then loaded onto a Ni-NTA column with FPLC system (GE HEALTHCARE) and eluted with a elution buffer (20 mM Tris-HCL, pH 7.5, 0.25 M imidazole, 0.15 M NaCl,) followed by a 100–400 mM imidazole gradient, after washed with 5 column volumes of washing buffer. The protein peak was further purified by size exclusion chromatography (Superdex 200 Increase column, GE HEALTHCARE) in 20 mM Tris-HCL, pH 7.5 and 0.15 M NaCl. Mutant proteins were purified using the same procedures. Selenomethionyl-derivative (SeMet) protein was obtained following well established protocols then purified and stored as described for the native protein^46–48^. The purified proteins were each concentrated to 20 mg/mL for crystallization screening.

### Crystallization, data collection, structure determination and refinement

All crystallization screening were performed using the sitting-drop vapor-diffusion method and Crystal Screen kit (HAMPTON RESEARCH). 1 μL SeMet-PcUP1 as well as PcUP1 and PcUP2 protein at 10 mg/ml was mixed with 1 μL reservoir and equilibrated against 150 µl of reservoir solution at 10 °C. Crystallization of SeMet-PcUP1 and PcUP1 was completed within 5 and 3 days respectively, in 0.1 M HEPES sodium (pH 7.5), 0.8 M NaH_2_PO_4_, 0.8 M KH_2_PO_4_. The PcUP1 crystals in complex with ligands were obtained by soaking crystals with mother liquor containing 10 mM each compound for 24 h. The crystals were directly mounted in a cryoloop and soaked with cryoprotectant solution (20% glycerol) and flash-frozen in cold liquid nitrogen prior to data collection. All X-ray diffraction data sets were collected at a wavelength of 0.97 Å on the beam line BL19U at temperature of 100 K in the Shanghai Synchrotron Radiation Facility (SSRF), China. The single-wavelength anomalous dispersion^48^ data were collected at the Se peak and remote wavelengths. The diffraction data from PcUP1 were collected with the 360° total rotation range, 1° per image, and with the “auto-correction” option in scaling. The “no merge original index” option was used to generate alternative, unmerged set of data, intended only to calculate correlation coefficient of anomalous difference for two random half set (CCano) by phenix.anomalous_signal. The data were scaled separately within 45°, 90°, 180°, 270°, 360° rotation range to analyze the strength of anomalous signal at different multiplicity. The statistics of diffraction data at maximal redundancy are listed in Table 1. The diffraction data were processed using HKL3000 software^49^. PcUP1 complexes using the structure of wild type PcUP1 as a search model were solved by molecular replacement (MR) with Phaser program^50^ from the CCP4 suite^51^. PcUP1 complexes using the structure of wild type PcUP1 as a search model were solved by molecular replacement (MR) with Phaser program^50^ from the CCP4 suite^51^. Further refinement was carried out by using the programs Phenix^52^ and Coot^53^. 5% of randomly selected reflections were used to calculate R free as a monitor. Subsequent refinements by incorporating ligands and water molecules were according to 1.0σ map level. The data collection and refinement statistics are summarized in Table 1. High-quality figures of the protein and ligand structures were created with the PyMOL program (http://pymol.sourceforge. net/).

**Table 1:** Data collection and refinement statistics of PcUP crystals.

	PcUP1 SeMet	PcUP1	PcUP1 URA/R1P	PcUP1 DUR/PO4	PcUP1 THM/PO4
**Data collectin**					
Space group	P222	P2_1_	p2_1_2_1_2_1_	p2_1_2_1_2_1_	P222
**Cell dimensions**					
*a*, *b*, *c* (Å)	51.38, 87.72, 119.472	67.24, 153.05, 67.28	66.975, 97.68, 188.865	66.790, 97.38, 188.520	66.956, 97.33, 188.346
β(°)	90.000	115.74	90.000	90.000	90.000
Resolution (Å)	50–2.17 (2.21–2.1)	50–1.57 (1.63–1.57)	50–2.5 (2.54–2.5)	50–2.10 (2.14–2.10)	50–1.97 (2.00–1.7)
*R*_merge_ (%)	22.9 (151.8)	15.9 (81.4)	15.3(55.9)	16.6(49.6)	19.9(98.4)
*I*/σ	13.54(1)	6.6(1)	20.7 (5.7)	16.9(6.7)	11.9(2.1)
Completeness (%)	99.9(100)	98.9(83.1)	100 (100)	99.9 (100)	99.7 (93.8)
Redundancy	13.3 (10.8)	3.4 (3.0)	13.3 (13.2)	13.9(13.3)	12.5(11.1)
**Refinement**					
Resolution (Å)	19.57–2.0 (2.2–2.0)	47.51–1.57 (1.63–1.57)	48.84–2.50 (2.59–2.50)	48.69–2.10 (2.17–2.10)	38.46–1.96 (2.03–16)
No. reflections	32841	144078 (5837)	43174 (4118)	72543 (7120)	87357(8200)
*R*_work_/*R*_free_	31.06 (24.18)/30 (23)	18.6(19.1)/21.7 (22)	26.1 (25.6)/34.8 (34.9)	24.62 (25.57)/31.7 (31.58)	26.5 (29.2)/31.56(32.85)
**No. atoms**					
Protein	4298	8814	8580	8606	8595
Ligand/ion	0	36	88	84	90
Water	197	1280	190	533	584
***B*-factors**					
Protein	19.3	17.3	39.7	30.2	29.7
Ligand/ion	-	13.7	31.9	23.9	27.3
Water	27.7	28.7	35.1	32.2	34.2
**R.m.s. deviations**					
Bond lengths (Å)	0.01	0.0062	0.01	0.0091	0.010
Bond angles (°)	1.17	0.84	1.15	1.06	1.20
**Ramachandran plot [%]**					
Most favored	95.79	96.7	94.0	94.5	95.43
Additionally allowed	4.21	3.07	5.45	4.64	3.87

Values in parentheses are for the highest-resolution shell. Data were collected from one crystal for dataset.

### Site-directed mutagenesis

Variants were constructed using a Fast Site-Directed Mutagenesis Kit (TIANGEN BIOTECH (Beijing) Co Ltd.) with the wild-type PcUP plasmid as a template. The sequences of the mutagenesis oligonucleotides are listed in Supplementary Table S1. The PCR products were incubated with DpnI (TIANGEN BIOTECH (Beijing) Co. Ltd.) to digest the original DNA template and then separately transformed into *E. coli* strain FDM competent cells. Each mutant site change has been confirmed by sequencing.

### Activity assays and substrate specificity

Assay mixture contained 3 mM uridine, 20 mM Tris–HCL (pH 7.5), and 10 mM Na_2_HPO_4_ at a protein concentration of 100 μg/ml. Control experiments were carried out in the absence of either enzyme or substrate in the assay mixture. All assays were performed at 25 °C for 10 minutes, then 10% of the reaction mixture was transferred to 900ul of MeOH to stop the reaction. To test if metal ions had an impact on enzyme activity, control experiments were carried out in the absence of EDTA or metal ions in the assay mixture, 2 mM EDTA, potassium chloride, calcium chloride, magnesium chloride were individually added into the assay mixture. After filtration through a 0.22-μm filter, 5 μL of assay solution was analyzed using a high-performance liquid chromatography system (HPLC, WATERS e2695, 2998 PDA Detector) equipped with a XBridge-C18 column (4.6 × 250 mm, 5 μm, Waters). The mobile phase was 5% (V/V) methanol at a flow rate of 0.8 mL min^−1^, and the effluent was monitored at a wavelength of 260 nm. The typical elution condition was 4–5 min with 5% methanol. The catalytic products of URA were identified by comparing the retention time of standard compounds. The peak areas of URA were calculated and presented as percentage of the wild type enzyme products. All samples were analyzed in triplicate in each independent experiment, from which the data were averaged and the standard errors were calculated.

Kinetic assays were performed over a range of uridine concentrations from 0.25 mM to 5 mM with seven replicates at each concentration using an assay time of 10 minutes. The Michaelis-Menten curve for each enzyme was fitted using the nonlinear regression model of GraphPad Prism 8 for Mac computers. K_cat_ values were estimated assuming an Et = 2.

### Accession numbers

The atomic coordinates and structure factors of the reported structures have been deposited in the Protein Data Bank under accession codes as follows: wild type of PcUP1, **6K5G**; DUR complex, **6K5K**; R1P complex, **6K5H***;* THM complex, **6K8P**. All other relevant data are available from the corresponding author upon request.

## Supplementary information

Supplementary Tables

Supplementary .Figure S1

Supplementary Figure S2

Supplementary Figure S3

Supplementary Figure S4

Supplementary Figure S5
