# Supplementary material for: Structural and catalytic analysis of two diverse uridine phosphorylases in Phytophthora capsici
Source: Sci Rep. 2020 Jun 3;10:9051. doi: 10.1038/s41598-020-65935-9 (PMC7271239; doi:10.1038/s41598-020-65935-9)
Supplement: Supplementary file 1 — Supplementary Tables [file 41598_2020_65935_MOESM1_ESM.doc]

Supporting information

**Structural and catalytic analysis of two diverse uridine phosphorylases in *Phytophthora capsici***

Cancan Yang^a,1^, Jing Li^a,1^, Zhenling Huang^a^, Xuefa Zhang^a^, Xiaolei Gao^a^, Chunyuang Zhu^a^, Paul F. Morris^b^, XiuGuo Zhang^a,*^

^a^Shandong Provincial Key Laboratory for Biology of Vegetable Diseases and Insect Pests, College of Plant Protection, Shandong Agricultural University, Tai’an, 271000, China.

^b^Department of Biological Sciences, Bowling Green State University, Bowling Green, OH,43403, USA.

^1^ Cancan Yang, Jing Li Contributed equally to the article

* Corresponding authors.

Phone: +86-538-8249095 (X. Zhang), Fax: +86-538-8249095 (X. Zhang), E-mail: [Zhxg@sdau.edu.cn](mailto:Zhxg@sdau.edu.cn) (X. Zhang).

**Table S1. Mutagenesis oligonucleotides**

|  | Sequence (5’ - 3’) |
| --- | --- |
| 505-H19D | GACGGCACCGTCCTC**GAT**TTGGGTCTGA |
| 505-E237K | ACACAGTCGAAATG**AAG**ACCTTTCACC |
| 505-Q206L | CCTTCTACAGCTCC**CTA**GGCCGTTTAGA |
| 505-R208D | TTCTACAGCTCCCAAGGC**GAA**TTAGACTCGAATTTTGAC |
| 505-T107A | ATTATCCGATTCGGG**GCG**TGTGGGGCTG |
| 505-R104E | GGACCGATGACCATTATC**GAA**TTCGGGACGTGTGGGGCT |
| 505-R59E | GAGACGTTCGAGTCAGCG**GAA**GGGTTCACAACGTATTCAG |
| 505-R39E | TCGGTTGGGAGTCTGGGG**GAA**GCCAAAGTGCTGGCTCAG |
| 505-R264E | GTACTGGTCGTAGCCAAC**GAA**CTCAGTGGCCAAATTGTTGA |
| 505-F202A | CTCAACGCTACCGCTTGCTCC**GCC**TACAGCTCCCAAGGCCG |
| 505-M80T | TTGTAGCGACAGGA**ACG**GGCGTCCCTAA |
| 505-N84I | AATGGGCGTCCCT**ATT**ATGGACTTT |
| 505-P83A | TCCATTGTAGCGACAGGAATGGGCGTC**GCT**AATATGGACT |
| 505-P83D | TCCATTGTAGCGACAGGAATGGGCGTC**GAT**AATATGGACT |
| 510-H32D | CTCGGACGTGCTGTTC**GAC**ATCGGACTCA |
| 510-E248K | GCGCAACATCGAGATG**AAG**GCGCGGCTG |
| 510-Q215L | GACTTCTACGAAGGC**CTG**GGCCGCCTG |
| 510-R217D | TTCTACGAAGGCCAGGGC**GAT**CTGGATGGTGCCATCTGC |
| 510-T140A | TACATTCGCATGGGC**GCG**AGCGGCGGC |
| 510-R137E | CACGGTGCCACGTACATT**GAA**ATGGGCACGAGCGGCGGC |
| 510-R93E | CCGATCGGCAGTACGTCG**GAA**TACACACTGTTCAAGGTG |
| 510-R63E | ACGGGCGGCAGCGCCGAA**GAA**ATGACCCACTTCGCGCAA |
| 510-R273E | TGCGTGACGCTGCTGAAC**GAA**CTGAACGGCGACCAGGTG |
| 510-F211A | GACGCTCAGCTGCAACGAC**GCC**TACGAAGGCCAGGGCCGC |

**Table S2. Primers used in the study**

| Primer | Sequence (5’-3’) | Restriction site | Usage |
| --- | --- | --- | --- |
| 505432-28a-F | CATGCCATGGGCATGGCTTACCAGAACACCAATG | *Nco1* | Cloning PcUP1 to PET28a (F) |
| 505423-28a-R | AAGGAAAAAAGCGGCCGCATCAAGTGGG GTGGAGACGAT | *Not1* | Cloning PcUP1 to PET28a (R) |
| 510891-28a-F | CATGCCATGGGCATGGCCCCCAACATGGACGCAGT | *Nco1* | Cloning PcUP2 to PET28a (F) |
| 510891-28a-R | ATAAGAATGCGGCCGCCGCGGACGCGTTGACCTTCGACT | *Not1* | Cloning PcUP2 to PET28a (R) |
| UBC-F | GTCGATCTGCTCACTGCTTAC |  | qRT-PCR of UBC gene (F) |
| UBC-R | CGCGCGGTCTGTTCTATAAAT |  | qRT-PCR of UBC gene (R) |
| PcUP1qRT-F | CGAGGGTTCACAACGTATTCA |  | qRT-PCR of PcUP1 (F) |
| PcUP1qRT-R | CCTTGTCTCCCGTACAACAAA |  | qRT-PCR of PcUP1 (R) |
| PcUP2qRT-F | ACGGTGCCACGTACATTC |  | qRT-PCR of PcUP2 (F) |
| PcUP2qRT-R | ACAGACTCCAGCTTGTTGTT |  | qRT-PCR of PcUP2 (R) |
