# Supplementary figures and images for: Structural and catalytic analysis of two diverse uridine phosphorylases in Phytophthora capsici
Source: Sci Rep. 2020 Jun 3;10:9051. doi: 10.1038/s41598-020-65935-9 (PMC7271239; doi:10.1038/s41598-020-65935-9)

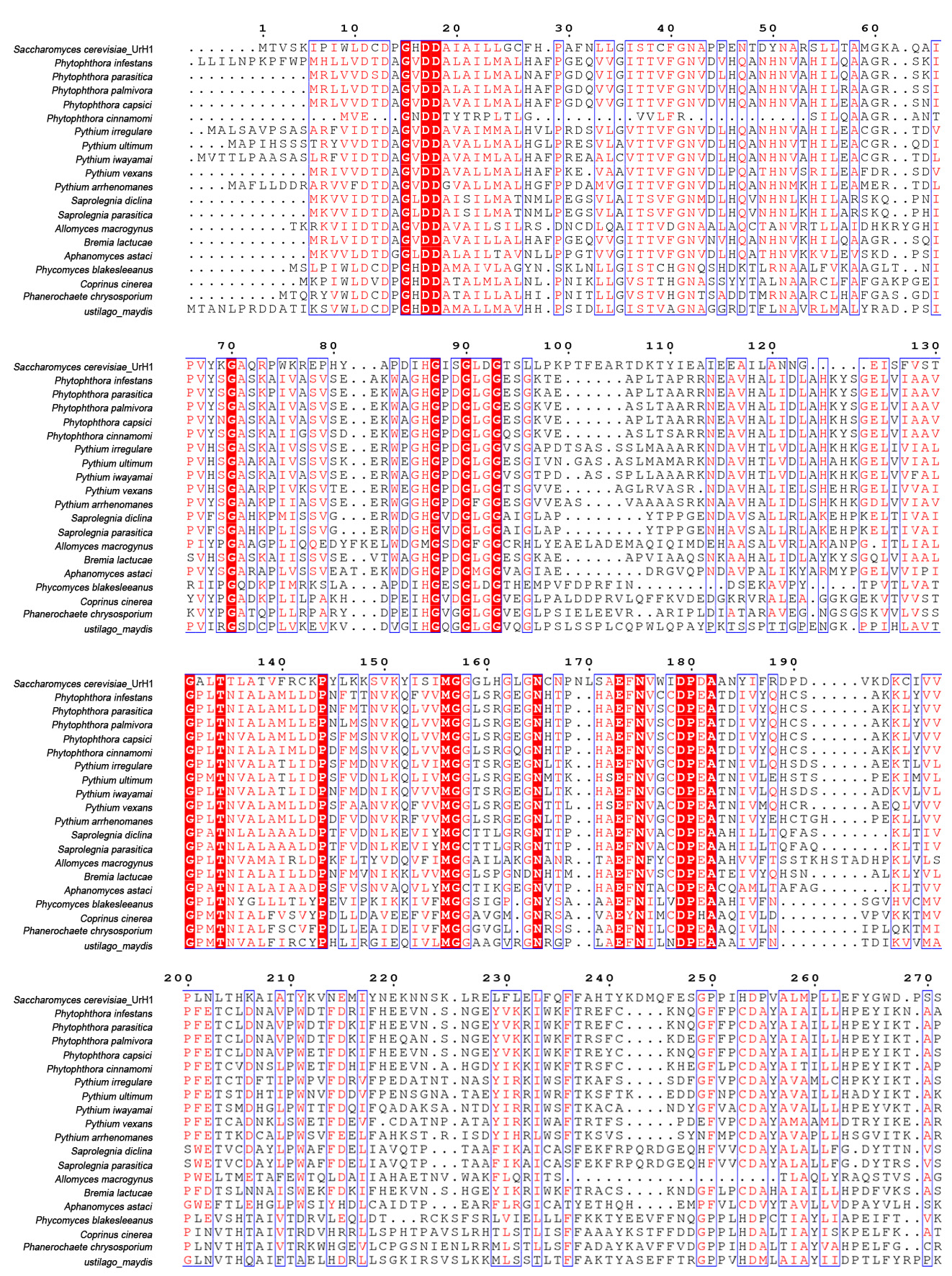

Supplement: Supplementary file 2 — Supplementary .Figure S1 [file 41598_2020_65935_MOESM2_ESM.jpg]

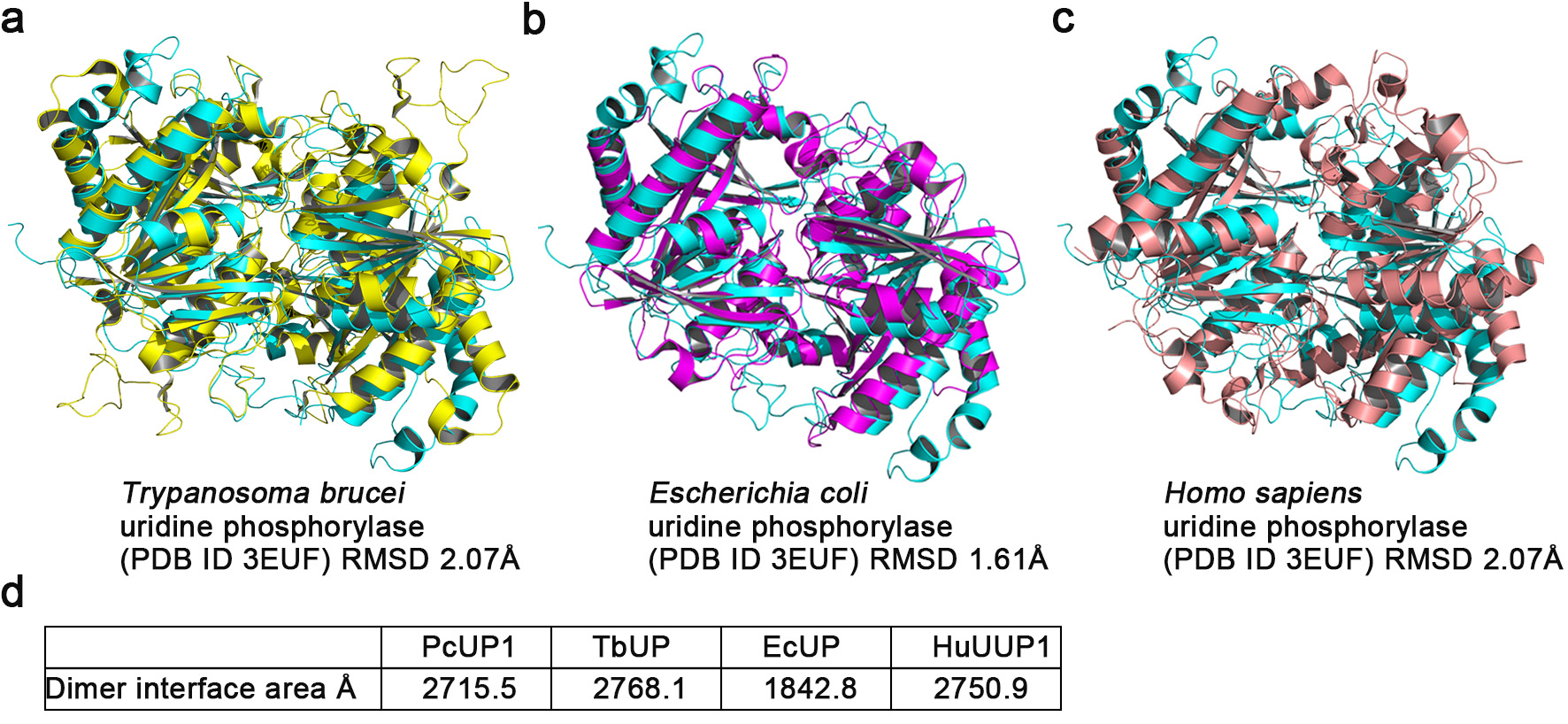

Supplement: Supplementary file 3 — Supplementary Figure S2 [file 41598_2020_65935_MOESM3_ESM.jpg]

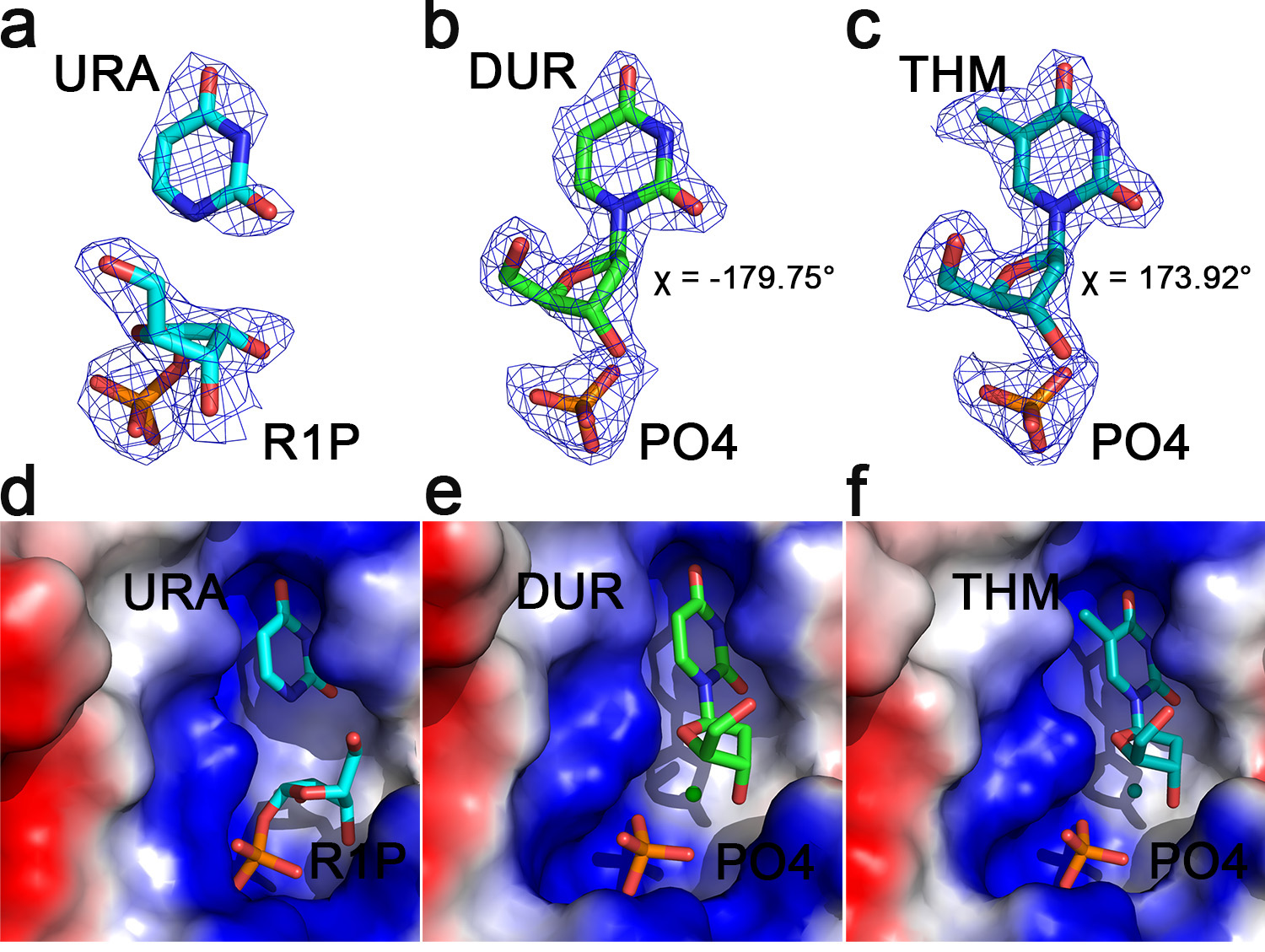

Supplement: Supplementary file 4 — Supplementary Figure S3 [file 41598_2020_65935_MOESM4_ESM.jpg]

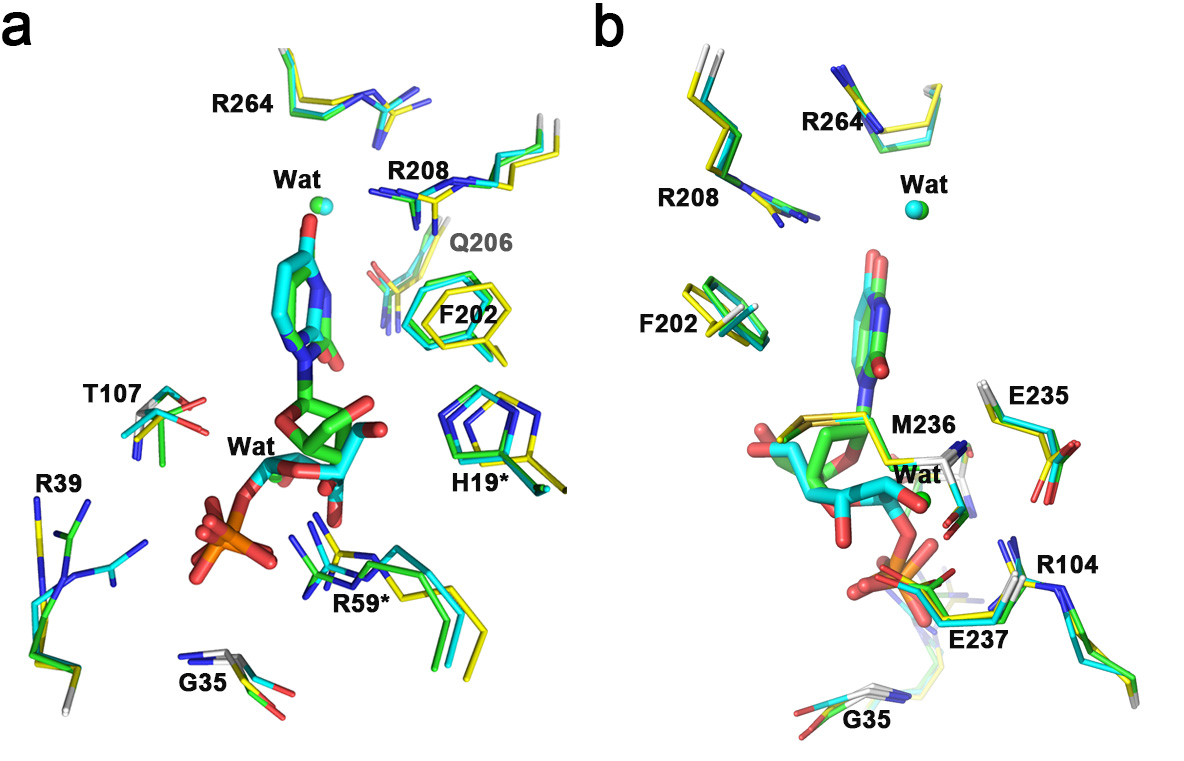

Supplement: Supplementary file 5 — Supplementary Figure S4 [file 41598_2020_65935_MOESM5_ESM.jpg]

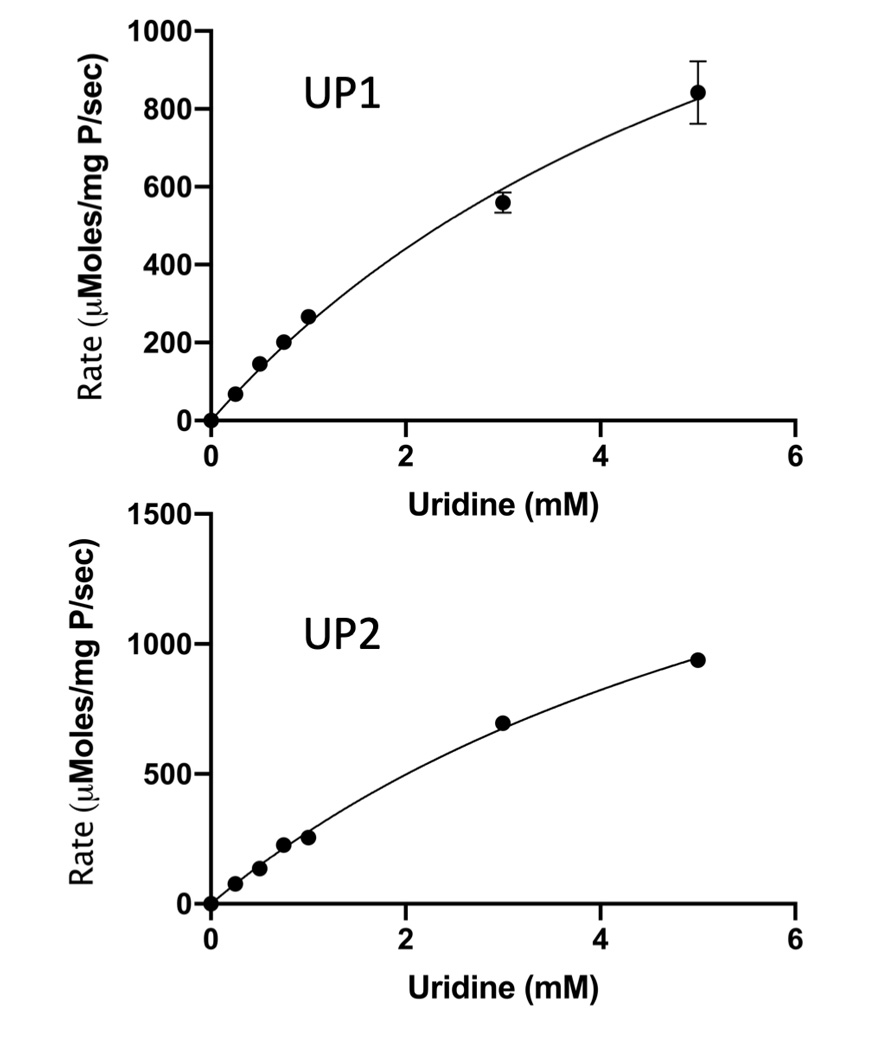

Supplement: Supplementary file 6 — Supplementary Figure S5 [file 41598_2020_65935_MOESM6_ESM.jpg]
